# Eco-Friendly Biosorbents Based on Microbial Biomass and Natural Polymers: Synthesis, Characterization and Application for the Removal of Drugs and Dyes from Aqueous Solutions

**DOI:** 10.3390/ma14174810

**Published:** 2021-08-25

**Authors:** Lăcrămioara Rusu, Cristina-Gabriela Grigoraș, Elena Mirela Suceveanu, Andrei-Ionuț Simion, Andreea Veronica Dediu Botezatu, Bogdan Istrate, Ioan Doroftei

**Affiliations:** 1 Department of Chemical and Food Engineering, Faculty of Engineering, “Vasile Alecsandri” University of Bacău, 157 Calea Mărăşeşti, 600115 Bacău, Romania; mirela.suceveanu@ub.ro (E.M.S.); asimion@ub.ro (A.-I.S.); 2 Department of Chemistry, Physics and Environment, Faculty of Sciences and Environment, “Dunărea de Jos” University of Galați, 111 Domnească Street, 800201 Galați, Romania; andreea.botezatu@ugal.ro; 3 Mechanical Engineering, Mechatronics and Robotics Department, Mechanical Engineering Faculty, “Gheorghe Asachi” Technical University of Iași, 43 Mangeron Blvd., 700050 Iași, Romania; bogdan_istrate1@yahoo.com

**Keywords:** biosorption, *Saccharomyces cerevisiae*, *Saccharomyces pastorianus*, alginate, chitosan, drug, dye

## Abstract

Pharmaceuticals and dyes are a very important part of the nonbiodegradable or hard biodegradable substances present in wastewater. Microorganisms are already known to be effective biosorbents, but the use of free microbial cells involves difficulties in their separation from effluents and limits their application in wastewater treatment. Thus, this study aimed to develop biosorbents by immobilizing *Saccharomyces cerevisiae*, *Saccharomyces pastorianus* and *Saccharomyces pastorianus* residual biomass on natural polymers (alginate and chitosan) and to evaluate the biosorptive potential for removal of pharmaceuticals and dyes from water. Six types of biosorbents were synthesized and characterized by Scanning Electron Microscopy and Fourier Transform Infrared Spectroscopy techniques and their biosorptive capacities for three drugs (cephalexin, rifampicin, ethacridine lactate) and two dyes (orange II and indigo carmine) were evaluated. The obtained results show that the removal efficiency depends on the polymer type used for the immobilization. In case of alginate the removal efficiency is between 40.05% and 96.41% for drugs and between 27.83% and 58.29% for dyes, while in the case of chitosan it is between 40.83% and 77.92% for drugs and between 17.17% and 44.77% for dyes. In general, the synthesized biosorbents proved to be promising for the removal of drugs and dyes from aqueous solutions.

## 1. Introduction

In the last few decades, due to the rapid industrialization and increasing of the world population, the ecosystems are seriously threatened due to the uncontrolled discharge of a large number of toxic organic or inorganic pollutants [1,2,3,4]. Among them, toxic metals, radionuclides as well as various organic pollutants, such as pesticides, pharmaceuticals, dyes, personal care products etc., can be listed [5,6,7].

Most of these inorganic and organic pollutants have accumulated in the environment due to their stability and difficult degradation and are detected in different environmental matrices: surface water, groundwater, soil and drinking water [2,3,8,9].

The World Health Organization states that water is an essential element for sustaining life, so water supply must be adequate, safe and accessible to all. Harmful effects on ecosystems and health hazards due to organic and inorganic pollutants present in water have been established over time, making it necessary to apply ever-higher standards for the detection and treatment of pollutants [10].

To remove pollutants from water, several physicochemical and biological processes have been developed such as adsorption, biosorption, coagulation, sedimentation, filtration, ion exchange, membrane technologies and biological treatments [11,12]. Some of these such as biological methods performed in wastewater treatment plants, are not effective in removing persistent organic pollutants [10].

Pharmaceuticals are a very important part of the nonbiodegradable or hard biodegradable substances present in wastewater and effluents from treatment plants. The annual production of pharmaceutical compounds has been estimated at thousands of tons worldwide [13]. Pharmaceutical residues have been detected in many environmental matrices around the world (e.g., in water, wastewater, sediment and sludge). The most important sources of pollution with these compounds are effluents from sewage treatment plants, hospitals and industrial units [14].

The presence of these pollutants in natural water poses a significant risk and some of them have already been declared as priority substances in water protection policies. Therefore, the development of innovative water treatment technologies is necessary to ensure water quality [15].

Lately, research has focused on removing persistent organic pollutants from water using as methods: adsorption/biosorption or advanced oxidation [11,16,17,18,19,20]. Biosorption is a relevant and increasingly studied process for the removal of pollutants. The literature shows that remarkable results have been obtained for the removal of persistent, inorganic and organic pollutants in low or medium concentrations in different types of aqueous effluents by using living and dead biomass, such as microorganisms, algae and agroindustrial waste [21,22].

This has been and remains a promising biotechnology for the removal of pollutants from aqueous solutions due to its simplicity, efficiency and to the availability of biomass and residual biomass. The biosorptive capacities of different types of biomasses in relation to the removal of different types of pollutants have been reported in numerous scientific papers [4,7,23,24,25,26].

Biosorption as a process is based on a variety of mechanisms including: adsorption, absorption, ion exchange, surface complexation and precipitation.

From the first research on the pollutants biosorption processes, the studies focused on obtaining efficient, viable, economical and easy-to-apply in wastewater treatment, biosorbents.

The literature presents numerous materials of biological origin that have been investigated to obtain biosorbents that include microbial biomass (bacteria, cyanobacteria, fungi, yeasts, microalgae), microbial residual biomass (resulting from fermentation processes), agricultural waste, food industry waste and other materials [10,23,27,28].

Both microorganisms and microbial residual biomass have been noted for good biosorptive capacity related to most pollutants tested. Still, they have a number of drawbacks, including the fact that free microbial cells are small in size and low in density, which involves difficulties in their separation from effluents and obviously, an increase in the cost of the biosorption process, which limits their application in wastewater treatment [10,23].

In this sense, the immobilization and encapsulation of microorganisms and residual biomass would be an alternative for obtaining biosorbents.

Different immobilization techniques and different supports were tested, among which we can mention polysulfone, polyvinyl alcohol, polyacrylamide, polyurethane, silica gel, carboxymethyl cellulose, brewer’s spent grain, sodium alginate, chitosan [4,7,10,23,24].

Natural polymers such as chitosan and alginate have been used successfully in the processes of immobilization/encapsulation of enzymes and pharmaceuticals [29,30,31]. So, studies have expanded to obtain adsorbents/biosorbents by immobilizing microorganisms or other materials [4,7,24,26,32,33,34,35,36,37,38,39,40,41].

Chitosan has been demonstrated that it can be used in adsorption processes because it is an inert, biocompatible, biodegradable and nontoxic material [7,32,41].

Alginate is also an alternative for immobilization due to its good chemical stability, nontoxicity, incorporation efficiency and low cost [4,7,24,26].

*Saccharomyces cerevisiae* is one of the most researched species in biosorption processes. Both *Saccharomyces cerevisiae* and *Saccharomyces pastorianus* are used in brewing and are available in large quantities.

In this context, this paper focuses on obtaining eco-friendly biosorbents that can be used in the treatment of effluents containing persistent organic pollutants.

This study aimed to develop biosorbents by immobilizing *Saccharomyces cerevisiae*, *Saccharomyces pastorianus* and *Saccharomyces pastorianus* residual biomass on natural polymers (alginate and chitosan) and to evaluate the biosorptive potential for removal of pharmaceuticals and dyes from water.

Six types of biosorbents were synthesized and characterized by scanning electron microscopy (SEM) and Fourier transform infrared spectroscopy (FTIR) techniques and their biosorptive capacities for three drugs (cephalexin (CPX), rifampicin (RIF), ethacridine lactate (EL)) and two dyes (orange II (O II) and indigo carmine (IC)) were evaluated.

The pharmaceutical compounds were chosen as target molecules taking into account the following considerations:

- CPX is an antibiotic which is considered an emerging compound with a risk factor greater than 1 in a number of countries [42];
- RIF is an antibiotic which is a persistent compound in water matrices with high potential to induce antimicrobial resistance in bacterial cells, leading to mutagenic and carcinogenic effects [22];
- EL is an antiseptic and local analgesic used in large quantities worldwide which can be found in effluents of wastewater treatment plants. Therefore it represents a potential risk for aquatic and other environments [43].

As for the selected dyes, both IC and O II are used in various industrial sectors (food, cosmetics, pharmaceuticals, textiles etc.), discharged in considerable amounts in water and recognized as recalcitrant compounds in water policies, difficult to remove by conventional water treatment [7,44].

Our literature survey revealed that RIF, EL, IC and O II are less studied from the point of view of their removal by adsorptive/ biosorptive processes. Therefore, the present study aims to provide new insights into application of these types of biosorbents in the removal of drugs and dyes from water. To the best of our knowledge, this is the first study to evaluate the biosorptive capacity of *Saccharomyces pastorianus* residual biomass immobilized on natural polymers related to removal of drugs and dyes from aqueous solutions.

## 2. Materials and Methods

### 2.1. Reagents and Analytics

All reagents implied in the experimental step were of analytical grade and were used without further purification. Sodium alginate and chitosan were bought from Carl Roth, Karlsruhe, Germany; sodium hydroxide and calcium chloride were delivered by Chempur, Piekary Śląskie, Poland; hydrochloride acid, sodium chloride, acetic acid, di-sodium hydrogen phosphate, potassium di-hydrogen phosphate, bentonite and ethanol were purchased from Chemical Company, Iași, Romania; cephalexin was from Cayman Chemical, Tallinn, Estonia; orange II and indigo carmine green were procured from Merck, Darmstadt, Germany.

Dried yeast strains of *Saccharomyces cerevisiae* and *Saccharomyces pastorianus* and residual biomass of *Saccharomyces pastorianus* were provided by two local companies (Rompak, Pașcani, Romania and Albrau, Onești, Romania).

Stock solutions of drugs and dyes with concentrations of 500 mg/L were prepared by dissolving the reagents in distilled water and kept at 4 °C in closed recipients.

For the calibration curves, adequate volumes of stock solutions were placed in series of 10 mL volumetric flasks. Concentrations ranges were between 1 mg/L and 30 mg/L for CPX; 1 mg/L and 150 mg/L for EL; 1 mg/L and 50 mg/L for RIF; 1 mg/L and 15 mg/L for O II and between 1 mg/L and 20 mg/L for IC.

Samples absorbance were acquired with the help of a UV1280 spectrophotometer (Shimadzu, Tokyo, Japan) at 260 nm for CPX, 431 nm for EL, 475 nm for RIF, 485 nm for O II and 610 nm for IC. Calibration graphs (absorbance vs. concentration) served to recover linear regression equations.

The solutions’ pH levels were adjusted with NaOH (0.1 M) or HCl (0.1 M).

### 2.2. Biosorbents Preparation

For *Saccharomyces cerevisiae*/calcium alginate (SC-A-5%) and *Saccharomyces pastorianus*/calcium alginate (SP-A-5%) beads preparation, aliquots of sodium alginate were placed in laboratory beakers containing hot water and mixed (Nahita heating magnetic plate (Auxilab, Beriain, Spain)) until complete dissolution. Dried yeasts strains were added to final concentrations of 5% on dry weight (d.w.) basis. The mixing process was continued until homogeneous suspensions were obtained. The resulting combinations were suspended in 2% calcium chloride solutions and kept at 4 °C.

*Saccharomyces cerevisiae*/chitosan (SC-C-2.5%) and *Saccharomyces pastorianus*/chitosan (SP-C-2.5%) beads were synthesized according to the method presented by Kaushal et al. [28] with some modifications. Briefly, homogeneous chitosan suspensions of 2.5% in 1% acetic acid (w/v) were prepared. In parallel, adequate amounts of dried yeasts and bentonite were dissolved into phosphate buffer (pH 7.5) and added to chitosan in order to reach final concentrations of 2.5% for yeasts strains (d.w.) and 0.05% for bentonite (d.w.). After complete homogeneity was attained, the mixtures were dripped in 2 M NaOH solutions, washed with phosphate buffer (pH 7.5) solution and stored at 4 °C.

For the biosorbents prepared with *Saccharomyces pastorianus* residual microbial biomass, the procedure used by de Rossi et al. [4] was considered. In a preliminary step, the biomass was submitted to multiple washing-decantation cycles. The recovered sediment was centrifuged for 20 min at 2500 rpm (Nahita 2615/1 centrifuge, Auxilab, Beriain, Spain) and then dried in a laboratory oven (AirPerformance AP60, Froilabo, Paris, France) for the determination of dried matter content, which was of 26.45%. Precise amounts of residual biomass (5% d.w. for SPRMB-A-5% biosorbent and 9% d.w. for SPRMB-A-9% biosorbent) were put in contact with 1% sodium alginate solutions in hot phosphate buffer (pH 7.0). The resulted homogeneous mixtures were suspended in 2% calcium solutions.

### 2.3. Biosorbents Characterization (Morphology, Functional Groups, Point of Zero Charge)

A Quanta 200 3D (FEI, Europe B.V., Eindhoven, The Netherlands) scanning electron microscope (SEM) with energy-dispersive X-ray system and a large field detector (LFD) was used for the surface morphology and elemental composition analyses of the synthesized biosorbents. The dried samples (50 °C, 2 h) were fixed to stubs with double adhesive carbon discs. Secondary electron mode (SE) in low vacuum was applied at an accelerating voltage of 20 kV, a working distance of 15 mm and a spot size of 5. The magnification range was between 1 mm and 10 µm.

Fourier transform infrared (FT-IR) spectra were registered between 4000 cm^−1^ and 400 cm^−1^ (32 scans coadded) with a resolution of 4 cm^−1^ on a Nicolet iS50 FT-IR spectrometer (Thermo Scientific, Dreiech, Germany) coupled with a built-in ATR accessory, DTGS detector and a KBr beam splitter. ATR ethanol cleaning was performed after each spectrum acquisition. The background spectrum reference with air was recorded and compared with the anterior one.

For the determination of biosorbents points of zero charge, the following modus operandi was applied. Volumes of 25 mL of 0.1 M NaCl solutions with initial pH values (pH_i_) settled between 2 and 12 with HCl (0.1 M) or NaOH (0.1 M) and measured with a portable pH meter (Dostmann KLH9.1, 0–14 pH, Carl Roth, Karlsruhe, Germany) were stirred on magnetic plates with 0.5 g of biosorbent for 24 h, at room temperature. Then, the pH values (pH_f_) were measured again. The pH_pzc_ were retrieved from curves pH_f_ = f(pH_i_).

### 2.4. Drugs and Dyes Biosorption

Samples of 25 mL of pharmaceuticals (CPX: 30 mg/L, pH 5; EL: 100 mg/L, pH 5; RIF: 50 mg/L, pH 6) and dyes (O II: 30 mg/L, pH 5; IC: 50 mg/L, pH 5) solutions were put in contact with 1 g of biosorbents beads for 12 h, at ambient temperature.

The residual concentrations were calculated by reading the samples absorbance at dedicated wavelength against the calibration curves.

The removal efficiency (*R*, %) and the biosorption capacity (*q*, mg/g) were determined with the Equations (1) and (2).

(1) $$R=\frac{C_{0}-C_{f}}{C_{0}}\cdot 100$$

(2) $$q=\frac{\left(C_{0}-C_{f}\right)\cdot V}{m}$$

where *C*_0_ and *C_f_* are the initial and final concentrations (mg/L); *V* is the sample volume (L) and *m* is the biosorbent amount (g).

## 3. Results and Discussion

### 3.1. Biosorbents Synthesis

For the biosorbents synthesis, two types of natural polymers, sodium alginate and chitosan were tested.

**Figure 1:**
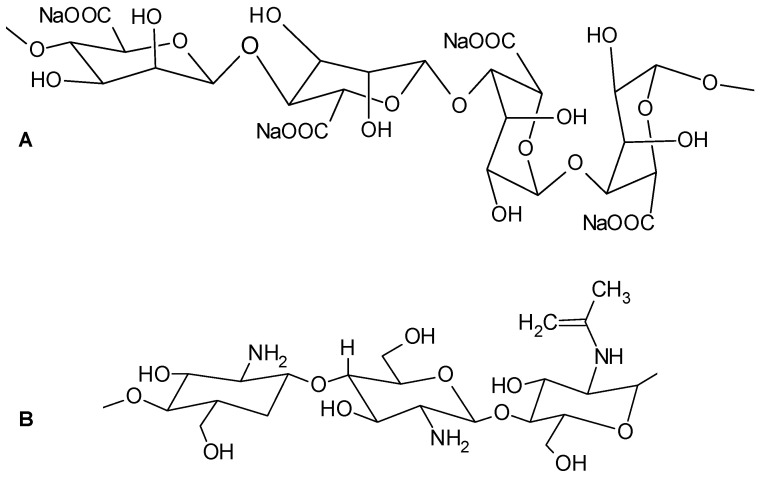
Molecular structure of sodium alginate (**A**) and chitosan (**B**) natural polymers.

Alginate (Figure 1A) is an alginic acid salt with a polymeric structure formed of blocks of α-L-guluronic acid and β-D-mannuronic acid linked by 1–4 glycosidic bonds [45] and which possess gelling properties. Various ions (hydrogen, calcium, aluminum, copper, zinc, iron, manganese etc.) can be responsible for alginate gelation but the most encountered one is the calcium cation. It interacts with guluronate units to form complexes which pair into dimers, which in turn associate to make multicomplexes [46]. Since this biopolymer is biodegradable, biocompatible and nontoxic, it is suitable for uses in processes such as formulation of pharmaceutical excipients [47], encapsulation of drugs or therapeutic agents [48,49], immobilization of different microorganisms [50,51], food packaging and stabilization [52] etc.

Chitosan (Figure 1B) is a polysaccharide consisting of units of D-glucosamine deacetylated and *N*-acetyl-D-glucosamine linked β-1,4. It is biocompatible, biodegradable and easily transformable in hydrogel [53] by emulsion methods, coprecipitation and crosslinking, extrusion crosslinking etc. [54]. It finds applications in sectors as drug delivery [55], encapsulation [56] or immobilization [57] as well.

In our study, these polymers were used to immobilize two different dried yeasts strains, *Saccharomyces cerevisiae* and *Saccharomyces pastorianus*, and one residual microbial biomass of *Saccharomyces pastorianus* issued from beer production.

**Figure 2:**
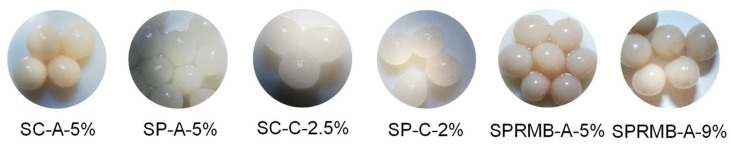
Biosorbents photographs.

The exchange of cations existing in the polymers with those of the collecting solutions ensure the development of crosslinkages in matrices chains causing bead formation. The procedure is affected by working parameters including pH, temperature, mixing velocity, polymer solution concentration, collecting solution concentration etc. As general rule, the biomass was incorporated homogeneously and uniformly in the polymeric matrices. Sphericity was attained for all the biosorbents. In all cases, the granules had smooth surfaces and opaque appearance (Figure 2). White nuances were observed for the obtained biosorbents, excepting the SPRMB-5% and SPRMB-9% which had a darker color explained by its residual components.

Diameters of the adsorbent materials were comparable with average values of 3.064 ± 0.074 mm for SC-A-5%, 2.921 ± 0.198 mm for SP-A-5%, 3.543 ± 0.128 mm for SC-C-2.5%, 3.231 ± 0.130 mm for SP-C-2%, 3.169 ± 0.163 mm for SPRMB-A-5% and 3.534 ± 0.074 mm for SPRMB-A-9%.

### 3.2. Biosorbents SEM Investigation

**Figure 3:**
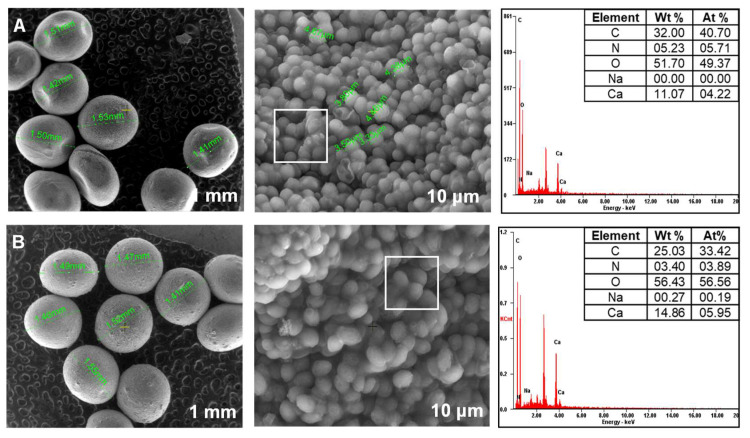
SEM images of biosorbents prepared by immobilization of yeast strains on calcium alginate polymer ((**A**): SC-A-5%; (**B**): SP-A-5%).

**Figure 4:**
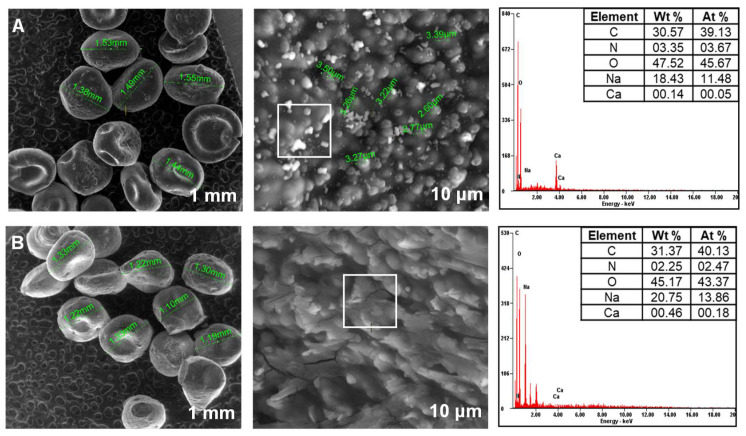
SEM images of biosorbents prepared by immobilization of yeast strains on chitosan ((**A**): SC-C-2.5%; (**B**): SP-C-2.5%).

**Figure 5:**
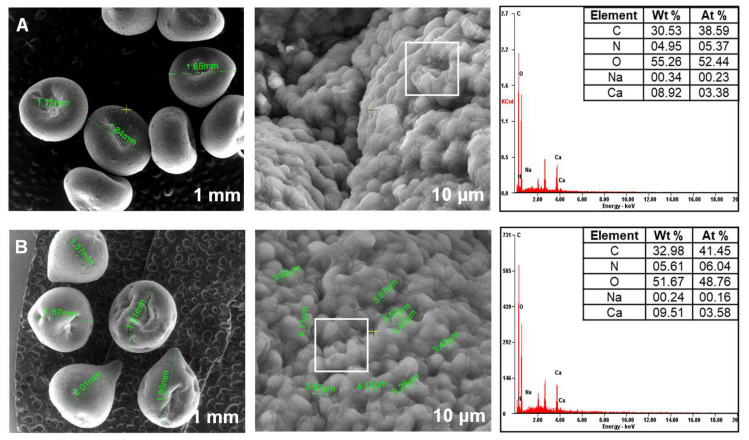
SEM images of biosorbents prepared by immobilization of residual microbial biomass on alginate ((**A**): SPRMB-A-5%; (**B**): SPRMB-A-9%).

Figure 3, Figure 4 and Figure 5 present images recovered by SEM analyses of the synthesized biosorbents. A loss of sphericity can be detected. It is less accentuated for the materials obtained by immobilization of *Saccharomyces cerevisiae* and *Saccharomyces pastorianus* on alginate (Figure 3) than for those on chitosan (Figure 4).

As expected, this behavior is caused by the water removal on drying and it has been described in other studies. Gholamian et al. [58] prepared and characterized calcium alginate hydrogels in which they incorporated cumin essential oil. Their morphology tests reveal that freeze-drying affected the beads shapes causing contraction and polymer crease. A considerable shrinkage after beads drying was remarked also by Voo et al. [59] who obtained calcium alginate beads with promising properties for drugs or foods encapsulation. Drying is also responsible for residual crystals of bentonite that can be observed on the beads’ surface of SC-C-2.5% (Figure 4A) and SP-C-2.5% (Figure 4B) materials.

In terms of elemental composition, similar proportions of carbon, nitrogen and oxygen exist in all the biosorbents. According to the preparation methods, differences in percentages of sodium and calcium are noted.

### 3.3. FT-IR Analyses of the Biosorbents

**Figure 6:**
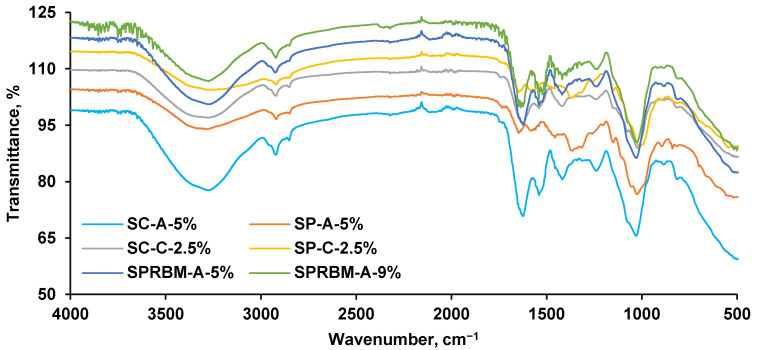
FT-IR spectra of the synthesized biosorbents.

FT-IR spectroscopy is often used to characterize materials and qualitatively identify the presence of various functions and groups. FT-IR spectra of the biosorbents developed in this research are depicted in Figure 6.

In SC-A-5% and SP-A-5% biosorbents obtained by immobilization of microorganisms on alginate, a CH_2_ bending vibration at approximatively 1020 cm^−1^ can be highlighted. At 1620 cm^−1^ and at 1418 cm^−1^, signals of the asymmetric carboxylic group can be noted while the band of 3270 cm^−1^ is specific for hydroxyl from alginate [60].

For SC-C-2.5% and SP-C-2.5% adsorption bands can be attributed as follows: 2920 cm^−1^ to CH_3_ symmetric stretch; 1646 cm^−1^ to N-H vibration (amide II) and 1576 cm^−1^ to C=O stretching (amide I) from chitosan but also specific for yeast cell walls constituents; 1363 cm^−1^ to CH_3_ vibration [32,61].

In SPRMB-A-5% and SPRMB-A-9% at 1318 cm^−1^ and at 1238 cm^−1^ stretching for amide III (proteins) and for asymmetric and symmetric PO^2−^ (phosphorylated proteins and phospholipids) were detected and were caused by the increased amount of yeast.

### 3.4. Biosorbents’ pH_PZC_

**Figure 7:**
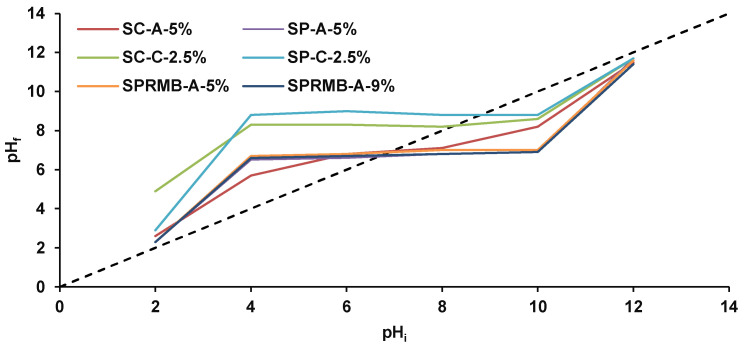
pH_PZC_ of the synthesized biosorbents.

Point of zero charge (pH_pzc_), defined as the pH value at which the biosorbents’ surface charge is null, i.e., the charge of the positive surface sites is the same as that of the negative ones, was determined with the drift method with sodium chloride as electrolyte. As shown in Figure 7, the shape is similar with an increase of pH_f_ with the augmentation of NaCl pH_i_, with a plateau when the pH_i_ is between 4 and 10 and with a new increase step after pH_i_ 10. The same evolution was mentioned by Kragovic et al. [62] who studied among others the influence of alginate encapsulation on the point of zero charge of several zeolites.

In our case, the recorded results are 6.9 for SC-A-5%, 6.6 for SP-A-5%, 6.9 for SPRMB-A-5% and 6.8 for SPRBM-A-9%, 8.3 for SC-C-2.5% and 8.8 for SP-C-2.5%. It can be seen that the type of biomass did not affect the pH_PZC_ values but they are dependent on the natural polymer used for the immobilization. For biosorbents prepared with sodium alginate, pH_PZC_ values were lower than those collected for biosorbent prepared on chitosan-bentonite. At pH below pH_PZC_, the biosorbents are considered positively charged while, when pH is superior to pH_PZC_, the biosorbents possess negative charge.

### 3.5. Biosorbents’ Applications to Drug and Dye Removal

**Figure 8:**
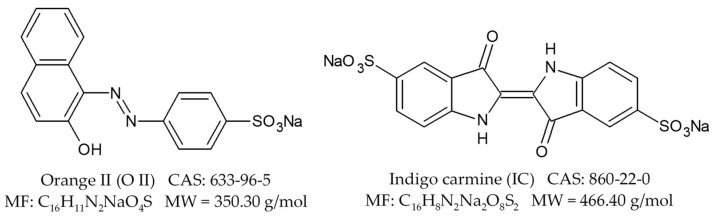
Dyes tested on the obtained biosorbents.

**Figure 9:**
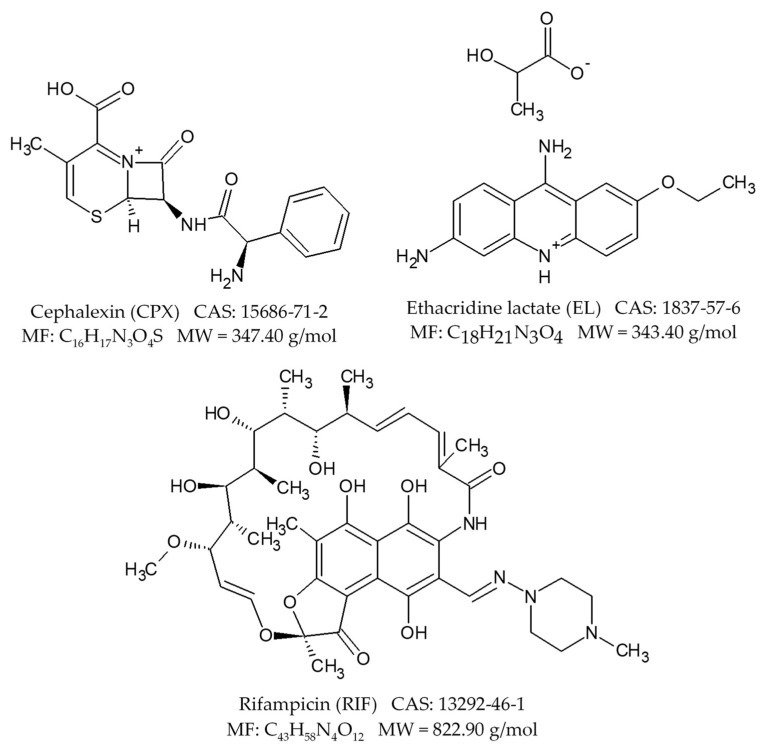
Drugs tested on the obtained biosorbents.

In order to examine the viability of the obtained biosorbents, we decided to test their capacity to retain pollutants. Therefore, three pharmaceuticals and two dyes were chosen as target molecules (Figure 8 and Figure 9).

**Figure 10:**
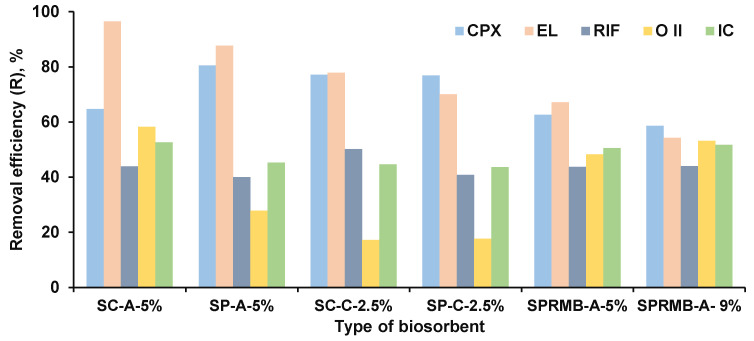
Removal efficiency of drugs and dyes on synthesized biosorbents.

Aliquots of 1 g of each biosorbent were put in contact with volumes of 25 mL of aqueous contaminants solutions. The pH values were set taking into consideration the adsorbates chemical structures and the biosorbents characteristics, especially their pH_PZC_. The calculated removal efficiencies are exhibited in Figure 10.

For the antibiotic cephalexin, the best biosorption capacities were recorded for the biosorbents prepared on chitosan (22.78 mg/g for SC-C-2.5% and 28.42 mg/g for SP-C-2.5%) and the lowest ones for those prepared by the immobilization of residual microbial biomass of *Saccharomyces pastorianus* (4.79 mg/g for SPRMB-A-9% and 9.26 mg/g for SPRMB-A-5%). Similar values were encountered when *Bacillus subtilis* bacterial biomass was used without treatment to remove CPX from aqueous systems [63,64]. As reported by Noman et al. [65], the retention mechanisms are electrostatic repulsion at pH below 4, electrostatic attraction, hydrophobic interactions, π-π bonds and Lewis acid-base interaction at pH between 4 and 6 and electrostatic repulsion and hydrogen bond at pH superior of 6. Moreover, between pKa values (2.56 and 6.88), the functional groups are negatively charged and CPX is in its molecular form fact that allows us to consider that at our working pH, the biosorption occurs by electrostatic attraction between the positive adsorbent surface and the zwitterionic form of CPX.

In the case of ethacridine lactate, the removal efficiencies were between 54.28% for SPRMB-A-5% and 96.40% for SC-A-5%. At present, very little information is available for the elimination of this molecule by adsorption. One of the publications dedicated to such study [43] reveals that the EL is well retained by activated carbons (in less than 60 min and at a concentration of 0.5 mM, more than 90% was eliminated from the solution).

Another of the tested molecules was the rifampicin, an antibiotic with a common structure consisting of a naphtohydroquinone chromophore spanned by an aliphatic ansa chain [66]. The recovered data show that the biosorption follows the same trend as in the case of CPX with the best values obtained for the biosorbents with chitosan as polymeric matrix (24.70 mg/g for SC-C-2.5% and 24.89 mg/g for SP-C-2.5%) while 1 g of SPRMB-A-5% was able to retain no more than 5.99 mg of pollutant. Electrostatic and π-π interactions and hydrogen bonding between RIF and the synthesized biosorbents may be responsible for the biosorption. Kais et al. [22] investigated RIF adsorption on a cocoa shell product and stated that the adsorption capacity was of 26.66 mg/g, while a hybrid nanomaterial prepared by Xu et al. [67] possessed an adsorption capacity of more than 90 mg/g for RIF found in solutions with concentrations between 3 mg/L and 20 mg/L.

Regarding the dyes’ biosorption, from Figure 10, one can observe that the results are also satisfactory. For O II, the removal efficiencies were of approximatively 18% for the biosorbents prepared by yeast strains’ immobilization on chitosan and significantly higher (more than 58% for SC-A-5%) for the other materials. For IC, the removal efficiencies present close and similar values for all the biosorbents (superior of 40% each time). The considered dyes are the subject of a wide variety of researches [7,44,68] which reveal that the adsorption process is due to electrostatic interactions and hydrogen bonding between the pollutants and the adsorbents.

In our tested conditions, the prepared biosorbents gave reasonable results in terms of the ability of removing drugs and dyes from aqueous solutions. However, it should be mentioned that in optimized settings, better uptake efficiencies could be expected.

## 4. Conclusions

The synthesized biosorbents exhibited similar characteristics, which proves that the techniques used for their synthesis are efficient.

The immobilization methods of microorganisms and residual biomass in the polymer matrix of alginate and chitosan were shown to be effective, the cells’ immobilization being confirmed by SEM and FT-IR analyzes.

The points of zero charge were identified at 6.9 for SC-A-5%, 6.6 for SP-A-5%, 6.9 for SPRMB-A-5% and 6.8 for SPRBM-A-9%, 8.3 for SC-C-2.5% and 8.8 for SP-C-2%. The pH_PZC_ values depend on the polymer type used for the immobilization and not the type of biomass that was used.

The obtained results show that the removal efficiency depends on the type of biosorbent and the concentration of the pollutant. In the case of using the alginate for immobilization the removal efficiency is between 40.05% and 96.41% for drugs and between 27.83% and 58.29% for dyes, while in the case of chitosan it is between 40.83% and 77.92% for drugs and between 17.17% and 44.77% for dyes. The biosorption capacities obtained in the presented test conditions vary between 4.79 mg/g and 85.42 mg/g for drugs and between 4.34 mg/g and 26.87 mg/g for dyes. This indicates a good biosorption capacity if we take into account the amounts of pollutants present in different water matrices.

The mentioned results reveal that all the synthesized biosorbents can be useful for the removal of pharmaceuticals and dyes from aqueous solutions. Further, by using the immobilization technique on natural polymers, first of all, the disadvantages of using free cells are eliminated and secondly, eco-friendly materials are obtained.

Finally, additional work is needed to study the effect of process parameters (pH, initial concentration, biosorbent dose and temperature) in order to optimize biosorption capacity.

## Data Availability

All data produced in this study are presented in this paper.
